# Supplementary material for: Risk of Liver Fibrosis Is Associated with More Severe Strokes, Increased Complications with Thrombolysis, and Mortality
Source: J Clin Med. 2023 Jan 2;12(1):356. doi: 10.3390/jcm12010356 (PMC9821417; doi:10.3390/jcm12010356)
Supplement: Supplementary file 1 [file jcm-12-00356-s001.zip › jcm-2064031-supplementary.pdf]

**Supplementary Table S1 – Frequency of AIS Outcomes per patient as analysed by FIB-4 cut-offs**

| | Total (n=887) | FIB-4 <1.3 (n=342) | 1.3≤ FIB-4 ≤2.67 (n=384) | FIB-4 >2.67 (n=161) | p-value |
|---|---|---|---|---|---|
| Functional Independence (mRS 0–2) (n, %) | 433 (48.8) | 213 (62.3) | 177 (46.1) | 43 (26.7) | <0.001 |
| 90-day Mortality (n, %) | 125 (14.1) | 21 (6.1) | 55 (14.3) | 49 (30.4) | <0.001 |
| SICH (n, %) | 47 (5.3) | 10 (2.9) | 21 (5.5) | 16 (10.0) | 0.004 |

Values are n (%) for categorical outcomes. Abbreviations: FIB-4 fibrosis-4, mRS modified Rankin Scale, SICH symptomatic intracranial hemorrhage

**Supplementary Table S2 – Post hoc Analysis of Baseline Characteristics of FIB-4 <1.3 vs. FIB-4 >2.67 group**

| **Pairwise median test between FIB-4 <1.3 and FIB-4 >2.67 group** | | |
|---|---|---|
| **Variable** | **p-value** | **Adjusted p-value** |
| Age | <0.001 | <0.001 |
| FIB-4 | <0.001 | <0.001 |
| BMI | 0.007 | 0.020 |
| HbA1c | 0.106 | 0.159 |
| LDL | <0.001 | <0.001 |
| HDL | 0.037 | 0.112 |
| Total cholesterol | <0.001 | <0.001 |
| Triglycerides | <0.001 | <0.001 |
| WBC | <0.001 | <0.001 |
| Lymphocytes | <0.001 | <0.001 |
| Neutrophils | 0.004 | 0.012 |
| Platelet | <0.001 | <0.001 |
| AST | <0.001 | <0.001 |
| Admitting NIHSS | <0.001 | <0.001 |
| Admitting SBP | 0.002 | 0.004 |
| **Pearson's chi-squared ($\chi^2$) test of homogeneity between FIB4 <1.3 and FIB4 >2.67 group** | | |
| **Variable** | **$\chi$2 test statistic** | **p-value** |
| Male | 10.156 | 0.001 |

| | | |
|---|---|---|
| Race (Chinese) | 11.902 | 0.001 |
| Race (Malay) | 2.7483 | 0.097 |
| Race (Indian) | 3.316 | 0.069 |
| Race (Others) | 1.8378 | 0.175 |
| Smoker | 20.07 | <0.001 |
| Hyperlipidemia | 0.37946 | 0.538 |
| Hypertension | 15.886 | <0.001 |
| Atrial fibrillation | 33.796 | <0.001 |
| Moderate to severe stroke (NIHSS ≥10) | 39.391 | <0.001 |
| Large vessel occlusion | 25.912 | <0.001 |
| Large-artery atherosclerosis | 2.0405 | 0.153 |
| Cardioembolism | 34.854 | <0.001 |
| Small-vessel occlusion | 23.256 | <0.001 |
| Stroke of other determined etiology | 1.0274 | 0.311 |
| Stroke of undetermined etiology | 0.18969 | 0.663 |
| 90-day mRS (0) | 25.149 | <0.001 |
| 90-day mRS (1) | 9.18 | 0.002 |
| 90-day mRS (2) | 4.3506 | 0.037 |
| 90-day mRS (3) | 0.2946 | 0.587 |
| 90-day mRS (4) | 3.0897 | 0.079 |
| 90-day mRS (5) | 7.5878 | 0.006 |
| 90-day mRS (6) | 53.932 | <0.001 |

*Abbreviations: FIB-4 fibrosis-4, BMI body-mass index, LDL-C low-density lipoprotein cholesterol, HDL-C high-density lipoprotein cholesterol, WBC white blood cell count, AST aspartate transaminase, NIHSS National Institutes of Health Stroke Scale, SBP systolic blood pressure, TOAST Trial of Org 10172 in Acute Stroke Treatment, mRS modified Rankin Scale*

**Supplementary Table S3- Baseline characteristics of participants as divided by SICH**

| | No SICH (n=840) | SICH (n=47) | p-value |
|---|---|---|---|
| FIB-4 (median [IQR]) | 1.50 [1.04, 2.25] | 1.96 [1.47, 3.16] | 0.001 |
| FIB-4 >2.67 (n, %) | 145 (17.3) | 16 (34.0) | 0.007 |
| FIB-4 <1.3 (n, %) | 332 (39.5) | 10 (21.3) | 0.019 |
| Age (years, median [IQR]) | 66 [56, 76] | 72 [63.5, 79] | 0.027 |
| Male (n/total, %) | 504 (60.0) | 24 (51.1) | 0.288 |
| BMI (kg/m$^2$, median [IQR]) | 24.44 [22.45, 27.34] | 25.00 [23.53, 27.68] | 0.364 |
| **Race** (n/total, %) | | | 0.224 |
| Chinese | 501/743 (67.4) | 34/43 (79.1) | |
| Malay | 155/743 (20.9) | 8/43 (18.6) | |
| Indian | 48/743 (6.5) | 0/43 (0.0) | |
| Others | 39/743 (5.2) | 1/43 (2.3) | |
| **Comorbidities** (n/total, %) | | | |
| Smoker | 125/840 (14.9) | 3/47 (6.4) | 0.161 |
| Hyperlipidemia | 438/840 (52.1) | 31/47 (66.0) | 0.090 |
| Hypertension | 555/840 (66.1) | 37/47 (78.7) | 0.103 |
| Diabetes mellitus | 262/506 (51.6) | 15/41 (36.6) | 0.092 |
| Atrial fibrillation | 168/840 (20.0) | 17/47 (36.2) | 0.013 |
| **Stroke parameters** | | | |
| Admitting NIHSS (median [IQR]) | 15 [8, 21] | 21 [18, 23] | <0.001 |
| Moderate to severe stroke (NIHSS ≥10) (n/total, %) | 574/833 (68.9) | 45/47 (95.7) | <0.001 |
| Admitting SBP (mmHg, median [IQR]) | 152 [136, 168] | 160 [146.5, 173.5] | 0.099 |
| Admitting DBP (mmHg, median [IQR]) | 82 [72, 92] | 82 [70.5, 90] | 0.754 |
| Onset-to-treatment time (min, median [IQR]) | 157 [118, 206] | 172 [139, 210] | 0.097 |

| | | | |
|---|---|---|---|
| Large vessel occlusion (n/total, %) | 508/773 (65.7) | 35/38 (92.1) | 0.001 |
| **TOAST (n/total, %)** | | | <0.001 |
| 1 | 280/821 (34.1) | 11/43 (25.6) | |
| 2 | 196/821 (23.9) | 22/43 (51.2) | |
| 3 | 136/821 (16.6) | 1/43 (2.3) | |
| 4 | 9/821 (1.1) | 2/43 (4.7) | |
| 5 | 200/821 (24.4) | 7/43 (16.3) | |
| **90-day mRS (n/total, %)** | | | <0.001 |
| 0 | 170/840 (20.2) | 1/47 (2.1) | |
| 1 | 184/840 (21.9) | 0/47 (0.0) | |
| 2 | 76/840 (9.0) | 2/47 (4.3) | |
| 3 | 92/840 (11.0) | 4/47 (8.5) | |
| 4 | 169/840 (20.1) | 10/47 (21.3) | |
| 5 | 44/840 (5.2) | 10/47 (21.3) | |
| 6 | 105/840 (12.5) | 20/47 (42.6) | |

*Values are median [IQR] for numerical variables & n/total (%) for categorical variables. Abbreviations: IQR Interquartile range, SICH symptomatic intracranial hemorrhage, FIB-4 Fibrosis-4, BMI body mass index, HbA1c hemoglobin A1c, LDL-C low-density lipoprotein cholesterol, HDL-C high-density lipoprotein cholesterol, AST aspartate transaminase, ALT alanine transaminase, WBC white blood cell count, NIHSS National Institutes of Health Stroke Scale, SBP systolic blood pressure, DBP diastolic blood pressure, mRS modified Rankin Scale, SICH symptomatic intracranial hemorrhage, TOAST The Trial of Org 10172 in Acute Stroke Treatment*

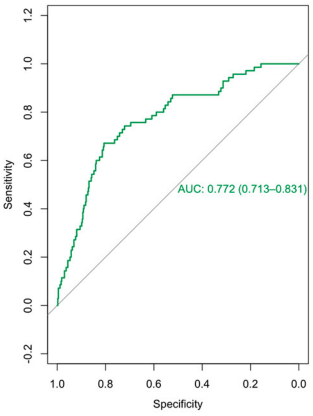


Supplementary Figure S1A. Receiver Operating Characteristic curve showing the classification performance and area under the curve (AUC) of the FIB-4 score for predicting mortality in a cohort of acute ischemic stroke patients undergoing thrombolysis. AUC was 0.772 (0.713-0.851)

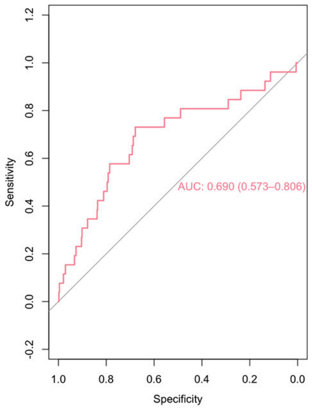


Supplementary Figure S1B. Receiver Operating Characteristic curve showing the classification performance and area under the curve (AUC) of the FIB-4 score for predicting symptomatic intracranial hemorrhage in a cohort of acute ischemic stroke patients undergoing thrombolysis. AUC was 0.690 (0.573-0.808)

**Supplementary Figure S1A and S1B – Receiving operating characteristic (ROC) curve evaluating predicting value of FIB-4 score in mortality and SICH respectively**
